# Antimicrobial, Antioxidant, and Anti-Inflammatory Properties of Monofloral Honeys from Chile

**DOI:** 10.3390/antiox12091785

**Published:** 2023-09-21

**Authors:** Erick Poulsen-Silva, Felipe Gordillo-Fuenzalida, Patricia Velásquez, Felipe M. Llancalahuen, Rodrigo Carvajal, Mauricio Cabaña-Brunod, María Carolina Otero

**Affiliations:** 1 Escuela de Química y Farmacia, Facultad de Medicina, Universidad Andrés Bello, Echaurren 183, Santiago 8370071, Chile; e.poulsensilva@uandresbello.edu (E.P.-S.); r.carvajalcorvaln@uandresbello.edu (R.C.); m.cabana@uandresbello.edu (M.C.-B.); 2 Laboratorio de Microbiología Aplicada, Centro de Biotecnología de los Recursos Naturales (CENBIO), Facultad de Ciencias Agrarias y Forestales, Universidad Católica del Maule, Avda. San Miguel 3605, Talca 3530000, Chile; fgordillo@ucm.cl; 3 Laboratorio de Compuestos Bioactivos, Departamento de Ciencias Químicas, Facultad de Ciencias Exactas, Universidad Andres Bello, República 330, Santiago 8370134, Chile; patricia.velasquez@unab.cl; 4 Laboratory of Integrative Physiopathology, Faculty of Life Sciences, Universidad Andres Bello, Santiago 8370134, Chile; fe.marchant@uandresbello.edu; 5 Millennium Institute on Immunology and Immunotherapy, Santiago 8331150, Chile

**Keywords:** bioactive compounds, chilean honey, anti-inflammatory, antimicrobial, antioxidant

## Abstract

Honey is a mixture of compounds produced by bees that has been appreciated by humanity since the creation of the oldest civilizations. It has multiple uses and can be a highly nutritional and healing substance. It has been used in traditional medicine as a natural alternative for the treatment of diverse clinical conditions. This is due to its reported bioactive properties. The objective of this article is to exhibit and analyze the biological properties of different types of honey originating from Chile based on their antimicrobial, antioxidant, and anti-inflammatory activities, focusing primarily on recompiling experimental studies made on monofloral honey of plant species present in the Chilean territory. The result of this bibliographical review shows that Chilean honey possesses remarkable bioactive properties, mainly antimicrobial and antioxidant activities, with a few studies on its anti-inflammatory activity. Most of these results were attributed to monofloral honey belonging to ulmo (*Eucryphia cordifolia*) and quillay (*Quillaja saponaria* Molina) plant species. These properties are related to the presence of several bioactive components, such as phenolic components (mainly flavonoids), hydrogen peroxide (H_2_O_2_), enzymes, proteins, and carbohydrates. The biodiversity of the flora and the environmental conditions of the Chilean territory are responsible for the wide range of bioactive compounds and biological properties found in Chilean honey. Further studies must be made to uncover the medicinal potential of these native honeys.

## 1. Introduction

Honey is composed of a mixture of compounds produced by bees (*Apis mellifera*) from the nectar of flowers. It is a natural product that has been used in ancestral medicine since the oldest civilizations, the most prominent being the Greeks, Romans, and Egyptians, as a part of their traditional medicine, which incorporates different ancestral practices based on cultural beliefs, theories, and historical records of said cultures. For this reason, different types of honey, some of them with scientific backing, are used in the maintenance of health as well as in the prevention and treatment of different illnesses [1].

Chile produces approximately 10,000 tons of honey per year, which represents 0.8% of the world’s honey production. Due to the fact that honey consumption in Chile is lower than the world average, it is mainly exported to other countries. This may be a promising business opportunity, as Chilean honey is produced from native plant species rich in phenolic compounds, among other interesting bioactive molecules that may provide beneficial biological attributes [2,3]. The Chilean flora is very diverse; therefore, each honey that originates from a specific geographical area could contain different properties attributed to native plants that can provide benefit for human health. Alongside the floral origin, other abiotic factors such as the geographical location and climatic conditions can also influence the biological properties of honey [4].

Honeys inherit their properties from the plant species used by bees as food. Therefore, the main characteristic of honey is its botanical and geographical origin. In this sense, Chilean honeys have a unique botanical origin in the world, given the high degree of endemism existing in Chile due to its location and geographical isolation: southern America, bordered to the north by the Atacama Desert, to the south by Antarctica, to the east by the Andes Mountain range, and to the west by the Pacific Ocean. Likewise, this location generates a wide range of climates in the country: desert in the north, tundra and glaciers in the east and south, humid tropical on Easter Island, Mediterranean climate in central Chile, subalpine continental in the mountain range, oceanic in the south, and polar climate in the Chilean Territory.

In Chile, honeys are classified according to a melissopalynological analysis, which determines the amount and type of floral pollen present in them [5]. Honeys with a frequency of floral pollen from a specific species greater than 45% are considered monofloral or unifloral and are named with the common name of the plant species. Honeys that present floral pollen grains from two species and whose sum is greater than 50%, with a difference of at least 5%, are called bifloral and are named after the two main plant species. In the case of those honeys that present floral pollens from several plant species, all of which have less than 45% frequency, they are called polyfloral or multifloral honeys. A subclassification of Chilean honeys by the origin of the plant species used by bees to collect them allows them to be differentiated as endemic/native, non-native, and mixed.

Ulmo (*Eucryphia cordifolia*), quillay (*Quillaja saponaria*), tebo o tevo (*Retanilla trinervia*), corontillo (*Escallonia pulverulenta*), arrayán (*Luma apiculata*), corcolén (*Azara celastrina*), avellano (*Gevuina avellana*), tiaca (*Caldcluvia paniculata*), tineo (*Weinmannia trichosperma*), peumo (*Cryptocarya alba*), chañar (*Geoffrea chilensis*), litre (*Lithraea caustica*), guindo santo (*Eucryphia glutinosa*), notro (*Embothrium coccineum*), tepú (*Tepualia stipularis*), maqui (*Aristotelia chilensis*) and sauce (*Salix humboldtiana*) have been documented in scientific literature.

**Figure 1:**
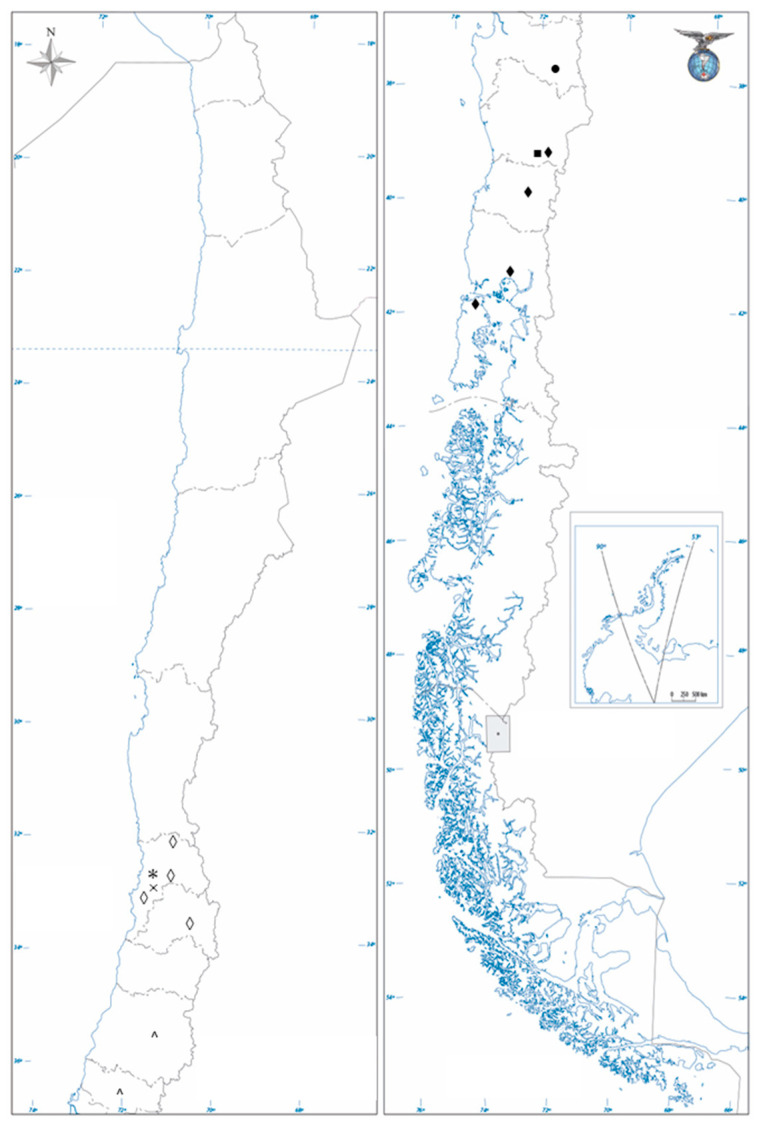
Locations of monofloral honey production. *E. cordifolia* honey is elaborated mainly in Villarica (39°16′55.1928″, Región de La Araucanía), Los Lagos (39°51′00″, Región de Los Ríos), Seno de Reloncaví (41°37′36.2454″, Región de Los Lagos), and Chiloé (42°36′0.003″, Región de Los Lagos) (♦). *Q. saponaria* honey is elaborated mainly in Petorca-Chincolco (32°14′33.1″, Región de Valparaíso), San Felipe (32°44′59.9634″, Región de Valparaíso), Lo Orozco (33°13′57.9″, Región de Valparaíso), and Río Clarillo (33°43′00″, Región Metropolitana de Santiago) (◊). *G. avellana* honey is elaborated in Santa Bárbara (37°39′51.7″, Región del Biobio) (●). The honey from *C. paniculata* is elaborated in Villarica (39° 16′ 55.1928″, Región de La Araucanía) (■). Honeys from *Azara* spp. are elaborated on in San Clemente (35°30′04.5″, Región del Maule) and Chillan (36°37′23.0″, Region de Ñuble) (^). The honey of *T. trinervia* Colliguay (33°11′21.2″, Región de Valparaíso) (×) (∗).

According to data obtained from the Oficina de Estudios y Políticas Agrarias (ODEPA), Chilean beekeeping is reported throughout Chile, with around 20,150 apiaries distributed from Arica y Parinacota to Magallanes and Antártica Chilena Regions [6]. Central and South Chile have a great number of apiaries and the production of honey, where a great diversity of flora is present, which is mainly native, endemic, and exotic [7]. The most studied in terms of biological properties are monofloral honeys from the nectar of the native *Eucryphia cordifolia* (Cunoniaceae), *Caldcluvia paniculata* (Cunoniaceae), species from the genus *Azara* (Salicaceae), endemic *Quillaja saponaria* (Quillajaceae), *Gevuina avellana* (Protoceae), and *Trevoa trinervia* (Rhamnaceae) species [7]. The locations of apiaries where the different monofloral honeys are elaborated are depicted in Figure 1.

In the current context, microbial resistance to antibiotics is an issue that has been of great concern over the years, generating a global alert since infectious diseases are one of the main causes of morbidity and mortality in the world. The excessive use of antibiotics worldwide, not only in a clinical scenario but also in the livestock industry, has led to the promotion of multiresistant bacterial strains that can withstand even the strongest antibiotics. In addition to this, the number of new antibiotics over the years has diminished, mainly due to pharmaceutical companies that ceased the development of new antibiotics because of economic and regulatory restraints [8,9]. That is why it is necessary to search for new alternatives that can support the treatment of multiresistant infections and cause the least damage to patients. It is known that honey has been part of traditional medicine, which uses products of natural origin such as plants, fungi, and algae in order to help with the treatment of various diseases caused by microorganisms or chronic pathologies. However, traditional medicine was discontinued due to the integration of synthetic agents in antimicrobial treatments [10]. Yet, the use of honey in current medicine is highly regarded for its important antibacterial properties that are used on infections caused by multidrug-resistant bacteria such as *Pseudomonas aeruginosa*, *Staphylococcus aureus*, and *Klebsiella pneumoniae*, since it possesses several effective bioactive compounds with bacteriostatic and bactericidal properties against a broad bacterial spectrum [11]. An important antibacterial and antifungal activity has been reported in ulmo and quillay honeys, the main honeys produced and exported by the country.

On the other hand, honey has also been shown to exhibit anti-inflammatory properties [1]. Inflammation is a regulatory biological process in our body that reacts against threatening stimuli such as allergens, infectious agents, or injuries. However, the constant exposure to several environmental, biological, and social factors may lead to the development of a chronic inflammatory response, which is linked to a considerable number of disorders, including allergies, metabolic syndromes, cardiovascular dysfunctions, cancer, and autoimmune diseases, which reflect on a huge health problem worldwide [12]. There are several treatments for regulating and suppressing the inflammatory crisis, mainly anti-inflammatory drugs, both steroidal and nonsteroidal in nature, and immunosuppressants. However, they can be associated with several adverse effects, including gastrointestinal ulcers, cardiovascular toxicity, alterations in hormone production, and other disruptions to normal biological functions [13,14]. For that reason, the main goal is to employ the minimum effective dose with the highest efficacy and fewer adverse effects. Therefore, it is necessary to consider the addition of natural anti-inflammatory components within medication therapy to help reach an increased pharmacological response with the lowest degree of undesirable side effects [15]. Recent works have reported the effective use of Chilean honey with applications on ulcers, with good results.

One final biological activity associated with honey is its high antioxidant properties, a mechanism that reduces the concentrations of reactive oxygen species (ROS) produced during an inflammatory process, which leads to high physiological stress that causes tissue damage [16]. High concentrations of ROS can also lead to the development of severe diseases of cardiovascular, muscular, metabolic, neurodegenerative, and carcinogenic nature [17]. The antioxidant capacity is attributed to the presence of phenolic compounds, especially flavonoids, due to their variable phenolic hydroxyl groups in their molecular structure, giving them the ability to both neutralize ROS and chelate metals such as iron. Flavonoids can also act upon biological enzymes such as lipoxygenase, cyclooxygenase, phospholipase A2, and NADPH oxidase, among others, further enhancing their antioxidant properties and acting upon other biological functions [18]. Chilean honeys present these antioxidant properties due to the content of phenolic compounds, mainly flavonoids, molecules that have a reducing power capable of donating a hydrogen atom to a free radical, neutralizing its reactive nature, and also due to the presence of vitamins and minerals (such as vitamin C, E, and selenium) that can scavenge and neutralize harmful free radicals [16,19]. Furthermore, some Chilean honeys present, in addition to these compounds, high mineral contents that would also contribute to their antioxidant capacity. In this sense, and derived from its composition, Chilean honeys present a wide degree of colors and aromas that give them attractive and unique sensory characteristics.

In this review, we presented a generalized bibliographical investigation of the properties of Chilean honeys, with emphasis on their antimicrobial, antioxidant, and anti-inflammatory properties evaluated in experimental studies, in order to demonstrate the multiple benefits of honeys originated in Chile, mainly those of monofloral origin. This information could be used to promote future studies of honeys from different geographical parts of Chile aimed at discovering their health benefits.

## 2. Material and Methods

The review was performed by analyzing scientific data published about Chilean honeys and their biological effects. The information was gathered using NCBI-Pubmed, Google Scholar, and Mendeley databases using the keywords: “Chilean honey” and “antioxidant property/activity”, “antimicrobial property/activity”, and “anti-inflamatory property/activity”. Scientific literature from 2005 to 2023 was used for this work.

## 3. Biological Properties of Chilean Honeys

According to the scientific literature consulted to date, Chilean honeys present biological properties such as antibacterial, antifungal, antioxidant, and anti-inflammatory characteristics.

### 3.1. Antimicrobial Properties

The antimicrobial properties of honey have been studied for many decades, demonstrating in several studies that it has both bacteriostatic and bactericidal properties. It is now known that each type of honey shows a different degree of antimicrobial activity due to the large number of factors that can alter the composition of bioactive components found in honey, such as enzymes, sugar content, peptides such as bee-derived defensin-1, and metabolic compounds [11]. A study showed that the bioactive components of honey are not the only factors that determine the different antimicrobial activity of each honey, but also factors such as the pH of the honey, the content of hydrogen peroxide (H_2_O_2_), and the osmotic pressure generated by honey on microorganisms [20]. The antioxidant capacity and the total flavonoid content also participate in the antimicrobial activity, showing that it probably depends on the synergic action of all its components to engage its activity against diverse microorganisms [21]. Currently, no cases have been reported in which microbial resistance to honey has been observed, suggesting that honey can be a candidate for future therapies against infections generated by pathogens that have generated resistance to antibiotics, which occurs much more frequently in chronic infections [22].

When it comes to monofloral honeys from Chile, a study using agar inhibition zone assays showed the antibacterial properties of different ulmo (*Eucryphia cordifolia*) honeys that also have a secondary pollen from another plant species. In these results, most of the samples showed activity against *S. aureus*, *Streptococcus pyogenes*, *P. aeruginosa*, and *Escherichia coli*; however, the sample that showed the greatest activity against the last two bacteria was the ulmo honey with secondary pollen from Alfalfa chilota (*Lotus pedunculatus*) and arrayán (*Luma apiculata*), respectively. A correlation was detected between the inhibition of *P. aeruginosa* and the presence of chlorogenic acid, as well as the inhibition of *E. coli* and the percentage of arrayán pollen [23]. Another study compared the antimicrobial capacity of various Chilean monofloral honeys, including ulmo, quillay (*Quillaja saponaria*), Chilean hazelnut (*Gevuina avellana*), and tiaca (*Caldcluvia paniculata*) honey. It was found that their methanol extract had a better antibacterial property compared to water dilutions, suggesting the important role that phenolic compounds could play in the antimicrobial activity of these honeys. This same comparative study also mentions the bactericidal activity of these extracts against the following common pathogens: *E. coli*, *P. aeruginosa*, *S. aureus*, and *S. pyogenes*, with minimal bactericidal concentration (MBC) results for all the honey types ranging from 3.1 to 6.1 g/L [24]. In a different study, phenolic extracts of two different samples of quillay honey presented antibacterial activities, which were observed on some of the bacteria mentioned above but also in other pathogens such as β-type *S. pneumoniae* and *Vibrio cholerae*. Its greatest in vitro activity was observed against β-type *S. pneumoniae*, with a minimal inhibitory concentration (MIC) result of 0.34 mg/mL in one sample (M338); and *S. aureus*, with a MIC result of 0.32 mg/mL in another sample (M337). [3]. One study compared the antimicrobial properties of ulmo honey and manuka (*Leptospermum scoparium*) honey against strains of methicillin-resistant *S. aureus* (MRSA), a pathogen attributed to the resistance of several antibiotics that have caused serious infections worldwide. Its results show that ulmo honey has a MIC of 3.1–6.3% *v*/*v*, compared to manuka honey with an MIC of 12.5% *v*/*v*, a result associated with the high content of H_2_O_2_ present in the honey extract, which enhanced its antibacterial effect [25]. Thus, it could be a promising candidate, particularly for wound infections caused by resistant pathogens to standard antimicrobial therapies.

Regarding the experimental studies made with multifloral honeys, a recent publication found that Gram-positive bacteria *Cutibacterium acnes* was susceptible to several samples of honey collected from different geographical locations ranging from the northern (Coquimbo), central (Valparaíso, Metropolitana), to the southern regions (Maule, Araucanía, Los Lagos, among others) of Chile, with a MIC of 9.4% *v*/*v* in 76% of the samples analyzed. In the case of the bacteria *S. aureus* and *S. epidermidis*, results showed a MIC of 37% *v*/*v* in 16% of the samples, and for *E. coli* and *P. aeruginosa*, these extracts reached MICs of 18.7% *v*/*v* in approximately 60% of the samples. These honey samples were compared with certified medical-grade honey (originated from manuka, ulmo, and tiaca) as an internal control, and it was observed that Chilean honey had antibacterial activity similar to the controls, showing MIC values of 18% and <9.4% *v*/*v* in all samples [21].

### 3.2. Antioxidant Properties

An antioxidant is a compound that can prevent the adverse effects of oxidation caused by free radicals, which can cause damage to various physiological functions if they are not controlled [19]. The antioxidant capacity of honey is directly related to the floral species from which the pollen is extracted, the environmental conditions in which the plant grew, and the harvest and post-harvest processing of honey. Furthermore, it was discovered that the honey polyphenol content varied with its color: darker colored honeys, classified as amber honeys, presented a significantly higher value of polyphenol content than the lighter colored honeys cataloged as white and extra white [20]. As well, it has also been found that high contents of minerals would also contribute to its antioxidant capacity [4,20].

A study that compared ulmo honeys that also had a secondary pollen showed that ulmo honey with pollen from tineo flowers (*Weinmannia trichosperma*) presented a significantly higher TPC than ulmo honey with other pollens such as arrayán or alfalfa chilota, with overall results ranging from 176–208 mg GAE/100 g honey (GAE: gallic acid equivalents). The antioxidant activity determined by 2,2-diphenyl-1-picrylhydrazyl (DPPH) radical assays showed that ulmo honey with arrayán as secondary pollen had better experimental results, with 152 mM eq Trolox/g honey [23]. In another study, monofloral honeys from species such as ulmo, quillay, tiaca, and Chilean hazelnut demonstrated an important antioxidant action evaluated by the ferric reducing activity power (FRAP) method, where honeys in water dilution presented a high antioxidant power due to their high presence of total phenols, with values between 848.7 and 1814.0 mg EAG/kg of honey (EAG: gallic acid equivalents). The highest antioxidant activity and total phenolic content (TPC) were attributed to quillay honeys from the town of Petorca-Chincolco, while the lowest TPC and antioxidant activity were described for a ulmo honey from Chiloe, although some ulmo honeys from different locations showed better antioxidant properties [24]. One study showed the antioxidant capacity of quillay honey extract using the oxygen radical absorbance capacity (ORAC) assay. Results of this study show that phenolic extracts derived from quillay honey have a lower antioxidant activity than the honey sample itself (0.72 ± 0.3 mmol Trolox eq/Kg and 1.5 ± 0.4 mmol Trolox eq/Kg of honey, respectively), based on the ORAC-PGR assay [26]. This suggests that an extract of phenolic compounds does not contain all the bioactive components with antioxidant activity that the honey extract has. It is mentioned that there are other components in the honey that have an antioxidant potential that contributes to the action that provides the polyphenolic compounds, thus explaining why honey has greater antioxidant capacity than the phenolic extract [27]. In another study, honey samples of corcolén (*Azara petiolaris and/orAzara integrifolia*), another native plant of Chile, showed an average TPC of 99.5 mg GAE/100 g honey, as well as an ORAC-PGR assay result of 0.33–4.49 µmol TE/g honey, proving to have antioxidant capacity [28]. A more recent study that evaluated multifloral honey harvested during 2012 and 2013 in different geographical locations in Chile showed that honey harvested in 2013 had antioxidant activity (average of 1.18 mmol Fe^2+^/100 g of honey for FRAP) equivalent to honey that is exported from Italy (0.06–1.2 mmol Fe^2+^/100 g of honey for FRAP) and New Zealand (0.6 mmol Fe^2+^/100 g of honey for FRAP). Moreover, it was demonstrated through the DPPH assay that the antioxidant content of honey presented great variability, especially according to the year of harvest and the geographical area of collection, with results of a half-maximal inhibitory concentration (SC_50_) of 56.89 mg/mL for the samples collected in 2012 and a SC_50_ of 44.69 mg/mL for samples collected in 2013. In addition, it was observed that the honey with the lowest variety of antioxidants was the one with the highest antioxidant activity, demonstrating that antioxidant activity does not depend on variability [21]. It is worth mentioning that a sample of Chilean multifloral honey had superoxide (O_2_^−^) scavenging activity comparable to that of manuka honey, with results of reciprocal IC50 (RIC_50_) of 59 mL/g and 48 mL/g, respectively [29].

### 3.3. Anti-Inflammatory Properties

Inflammation is a condition that occurs in response to trauma or infectious agents that cause damage to a tissue of the human body; therefore, it is a defensive form of response of the human organism to eliminate any foreign agent that may infiltrate through the damaged tissue [30]. It has been shown for several years that honey has anti-inflammatory activity; however, this action is carried out by several molecular mechanisms, mainly through the inhibition of complement activation, inflammatory cell infiltration, cytokine production, macrophage phagocytosis, and nitric oxide production [31].

One comparative study between seven different honeys, among which was a Chilean multifloral honey from the Maule region, discovered that said honey has an anti-inflammatory activity similar to manuka honey, which is well known for its attributes and its high anti-inflammatory capacity, making Chilean honey a possible alternative to manuka honey. The anti-inflammatory activity presented by Chilean honey is based on the inhibition of the generation of ROS produced by polymorphic nuclear cells (PMN). These are small, highly reactive molecules that are produced as a defense mechanism against pathogens that may be found in the damaged tissue area, yet long-term exposure to ROS can cause cell damage and inhibit wound healing. Despite these results, the manuka honey surpassed it in its activity of inhibiting the classical complement pathway, to which the multifloral Chilean honey scored a lower inhibitory activity. The complement system allows the recruitment of pro-inflammatory mediators in the area of damage, therefore inhibiting a fundamental step in the inflammatory process [29]. Yet, ROS can also increase the activation of nuclear factor-kappa B (NF-κΒ), which is known to be a factor that promotes the expression of pro-inflammatory mediators such as TNF-α and interleukins (ILs) such as IL-1b, IL-6, and IL-23 [32]. Therefore, inhibition of ROS generation would reduce the expression of pro-inflammatory mediators by reducing inflammation at the site of damage. Ultimately, a study carried out with Chilean ulmo honey showed that this honey supplemented with ascorbic acid could be a good treatment for wounds, generating a control of local inflammation in addition to rapid healing in the area of application since it increases the production of human keratinocytes, fibroblasts, and endothelial cells, thus accelerating the re-epithelialization of the damaged tissue [33].

Because of this, honey exhibits anti-inflammatory properties through several molecular mechanisms. First, its rich content of bioactive compounds such as flavonoids and polyphenols can inhibit the production of pro-inflammatory signaling molecules, such as cytokines and chemokines, by interfering with various intracellular pathways involved in inflammation. Second, honey’s low pH and high sugar content can create a hostile environment for bacteria, reducing the risk of infections that can trigger inflammation. Additionally, honey’s ability to scavenge free radicals due to its antioxidant constituents helps mitigate oxidative stress, a key driver of inflammation [16].

The results of all studies on the biological activity of honeys are summarized in Table 1, primarily of monofloral origin.

## 4. Discussion

With all the evidence reported and collected, it is clear that Chilean honey has remarkable biological activity. Their antimicrobial, antioxidant, and anti-inflammatory properties show a promising opportunity for further research in the field of bioactive products of natural origin. Yet, it is also important to verify if the honey type influences its general properties. Monofloral honey has been mainly praised due to its quality and commercial value [29], yet it was also observed that multifloral honey also possesses some important bioactive properties, despite the apparent lack of further study [21]. Most monofloral honey from Chile is harvested from the central and southern zones of the country, incorporating a climatic transition between the Mediterranean and humid temperate climates, which further enhances the region’s biodiversity. Some examples of species include the quillay from the central zone and the ulmo from the southern zone of Chile [25].

The ulmo tree is a member of the Cunoniaceae family, native to the central south of Chile, and is widely appreciated for its wood, ornamental characteristics, and quantity of nectar it produces [35]. Acevedo et al., 2017, investigated the presence of volatile and non-volatile components in ulmo honey, and they identified 50 volatile and 27 non-volatile compounds in the honey, with compounds such as benzaldehyde, ethyl benzoate, linalool, and *p*-anisic acid that have been partly associated with antibacterial properties [35]. Phenolic acids such as coumaric, gallic, chlorogenic, and caffeic acids have been found in samples of ulmo honey, as well as abscisic acid, pinocembrin, chrysin, quercetin, luteolin, apigenin, and rutin. Many of these polyphenols are known to have bioactive properties, such as antimicrobial, antioxidant, anti-inflammatory, and anticancer activities. Although some of these compounds have also been found in other monofloral honeys such as quillay and pine honey, ulmo honeys have been reported to have a higher TPC in several studies recompiled by Velásquez et al., 2019, compared to other honeys such as sesame, rosemary, orange, chestnut, blackberry, honeydew, and manuka, to name a few [23]. Among the most recent investigations, it has been verified that the biological activity of polyphenols that were previously mentioned contributes to the great properties that this type of honey possesses [36,37]. This is how the anti-inflammatory and antimicrobial effects are related to the gallic acid present in samples of this honey, while compounds such as rutin, chrysin, and pinocembrin have demonstrated anti-inflammatory and antimicrobial activities, thus making ulmo honey a natural substance with multiple properties, and among all those that exist in Chile, being the most studied and cataloged as the strongest within their activities [23].

The quillay tree belongs to the Quillajaceae family and is found mainly in the central zone of Chile, due to its preference for Mediterranean climates. Its bark, leaves, and flowers have been traditionally used in Chile by the Mapuche people as a detergent capable of forming foam in contact with water and also as a medicinal herb, for which its infusions and decoctions have been attributed to expectorant, anti-inflammatory, analgesic, and diuretic uses [38]. The different phytochemicals that occur in the quillay plant can produce honey with an antioxidant activity comparable to that of multifloral honey. The contribution of each compound is not known; however, the great variety of these can probably contribute to the total antioxidants that this honey possesses [4]. Regarding the exact composition of quillay honey, studies have shown that it contains various phytochemicals such as chlorogenic acid, caffeic acid, p-coumaric acid, vanillic acid, salicylic acid, abscisic acid, ferulic acid, kaempferol, hesperidin, aesculetin, quercetin, naringenin, and rutin [3,39].

Based on the previously recompiled studies, most of these are focused on the bioactive properties of ulmo and quillay honey. If we compare both monofloral honeys, we find that ulmo honeys prove to have a better antimicrobial capacity compared to quillay honey, where lower quantities of ulmo honey exert a superior antibacterial effect on pathogenic species such as *E. coli* and *S. pyogenes* [24]. Ulmo honey also showed activity towards clinical strains of MRSA, and compared to manuka honey, Chilean honey surpassed it in its antibacterial effect [25]. However, said activity is associated with its high content of H_2_O_2_, a factor that is considered not optimal in some studies. The antibacterial properties of honeys, which depend on many factors, have been defined into two types: peroxide and non-peroxide. Most honeys possess peroxide capacity; however, some honeys, such as manuka, have non-peroxide components that have been attributed to their remarkable biological effects and have set unique quantification units to determine their aforementioned properties. Methylglyoxal (MGO) is one of the main non-peroxide components identified in manuka honey, considered a standard within the biological properties of said honey. On the other hand, H_2_O_2_ is produced in honey due to the enzymatic action of glucose oxidase, adding to its antimicrobial potential; however, it can be easily neutralized by heat or by other enzymes such as catalase. It is why non-peroxide activity is more valued in some studies [33,40]. In the case of Chilean honeys, Montenegro et al., 2021, found that some ulmo honey retained its antimicrobial activity after the removal of H_2_O_2_, which was attributed to the presence of non-peroxide components in the honey [41]. Some quillay honeys also showed a certain activity to different bacteria [3]; however, this kind of honey showed to possess a higher antioxidant capacity compared to ulmo honey [24], having a remarkable TPC and antioxidant activity by itself, based on different experimental assays [26,34].

Chilean multifloral honeys also showed to have an important bioactive capacity. In the study of Olate-Olave et al., 2021, we can observe that multifloral honey can have exceptional antioxidant properties, as well as high phenolic content and antimicrobial activity [21], since multifloral honeys have a chance of possessing a wider variety of pollen. This type of scenario can also apply to the presence of secondary pollen in monofloral honeys. In the case of ulmo honey, it was observed that the presence of secondary pollen from native species such as tineo and arrayán can greatly increase the honey’s antioxidant properties and TPC. In the antibacterial assays, the ulmo honey that also contained alfalfa chilota pollen had superior activity against bacteria such as *S. pyogenes* and *P. aeruginosa*. The presence of compounds such as chlorogenic acid, chrysin, and apigenin can also be related to the different secondary pollens present in the honey, as well as the main honey itself [23]. It is clear that when it comes to the bioactive properties of honey, either monofloral or multifloral, it all depends on two major factors: the floral origins of the honey and the environmental conditions in which it is produced. The floral source of honey can greatly determine its chemical composition and bioactive properties. Some compounds have been found in specific types of monofloral honeys and have been used as biomarkers for their identification, such as manuka honey or the presence of the alkaloid caffeine in monofloral honeys of *Coffea* spp. [42]. The geographical location and climatic conditions of Chile have led to a high level of endemism in its ecosystem, with several diverse species of floral origin. Since honeybees are very selective in the floral sources for their honey, native species from Chile showed that they met the nectar requirements that bees favor, both in quantity and chemical composition, leading to the production of honeys with unique characteristics [22]. Native and endemic flora such as quillay and ulmo have led to a high-quality monofloral honey with evident bioactive properties. And with multifloral honeys from Chile, it is plausible that their properties could also be as remarkable as monofloral honeys, since there is a high chance some pollen from these native plants may be present in the honey.

Finally, regarding the comparisons made between Chilean honeys and honeys from different countries, Sherlock et al., 2010, exerted their comparisons with the aforementioned manuka honey, and their results showed that ulmo honey had lower MIC values than manuka honey in agar diffusion assays with MRSA. The authors of said study declared that these results may not be significant due to previous experimental reports of manuka honey, where they associated variations in the antimicrobial results based on the sensitivity of the bacterial strains and the potency of the manuka honey, which is proportional to the quantity of MGO [25,43]. Van der Berg et al., 2008, compared different samples of honey, which included multifloral honey from Chile, manuka honey, buckwheat honey (*Fagopyrum esculentum*), macadamia honey (*Macadamia integrifolia*), kiawe honey (*Prosopis pallida*), and a mixture of clover (*Trifolium* spp.) and alfalfa (*Medicago sativa*) honey from Canada, evaluating their in vitro antioxidant and anti-inflammatory properties on PMNs. Their results showed that despite the Chilean honey having activity, buckwheat honey, which has a low pH, high free acid content, and high sugar content and osmotic value, surpassed it in all aspects. In this sense, Chilean multifloral honey managed to have better antioxidant properties than macadamia, kiawe, and Canadian honey, based on the results of superoxide anion scavenging and inhibition of ROS produced by PMNs [29]. There is no doubt that honeys originating from Chile have remarkable biological properties, both multifloral and especially monofloral. Despite the lack of certainty regarding the results of their properties when compared to other bioactive honeys with validated backing, it is clear that with further experimental studies, we can reevaluate their importance in the investigation of bioactive honey that can bring a new look into natural bioactive compounds and alternative therapies.

## 5. Conclusions

Most studies concluded that Chilean honeys have good anti-inflammatory, antioxidant, and antimicrobial properties compared to other international honeys, which positions them as a good alternative for future research and marketing. This is attributed to the large and unique diversity of flora in the Chilean territory, which provides various types of honey with exquisite biological properties and therapeutic value. The biological activities that these honeys present have been mainly studied in vitro, giving a possibility for future in vivo studies that can evaluate the properties of native Chilean honeys in more complex ways and further validate them as an adjuvant for antimicrobial treatments, chronic inflammatory diseases, and in some conditions where high expression of ROS occurs. The biochemical results based on in vitro analysis clearly demonstrate the potential role of different Chilean honeys in inhibiting proinflammatory mediators as well as their antioxidant activity, demonstrating their high ability to reduce highly oxidizing compounds such as H_2_O_2_ or O_2_^−^ radicals, which are involved in a significant reduction in the quality of life of people worldwide. However, there is a lack of research on possible chemical markers, their performance on biological properties, and their possible synergistic effects. Finally, honey from Chile was shown to inhibit the microbial growth of antibiotic-resistant bacteria such as MRSA and act against common pathogenic bacteria such as *E. coli*, *S. aureus*, and *S. pyogenes*, to name a few, showing that these types of honeys have great potential in the research of treatments against multiresistant bacteria.

## Figures and Tables

**Table 1:** Summary of the bioactive properties of various Chilean honeys.

Honey Type	Antimicrobial Activity	Antioxidant Activity	Anti-Inflammatory Activity	Proposed Chemical Markers	References
*Eucryphia cordifolia* (ulmo)	*Pseudomonas aeruginosa*, *Streptococcus pneumoniae type β*, *Vibrio cholerae*, *Candida albicans*, *Escherichia coli*, *Salmonella typhi*, *Staphylococcus aureus*, *Haemolitic Streptococcus*	Determined by TPC, DPPH, and FRAP assays	Reported wound healing properties in association with anti-inflammatory mechanisms	Gallic acid, caffeic acid, p-coumaric acid, pinocembrin, chrysin, quercetin, luteolin, apigenin, chlorogenic acid, salicilic acid, esculetin, escopoletin, isoforone, cetoisoforone, trans β-damascenone, rutin	[23,24,25,33]
*Quillaja saponaria* (quillay)	*Pseudomonas aeruginosa*, *Streptococcus pneumoniae* type β, *Vibrio cholerae*, *Candida albicans*, *Escherichia coli*, *Salmonella typhi*, *Staphylococcus aureus*, *Haemolitic Streptococcus*	Determined by TPC, DPPH, FRAP, and ORAC assays	None reportem	chlorogenic acid, caffeic acid, syringic acid, rutin, escopoletin, p-coumaric acid, vanillic acid, salicilic acid, gallic acid, ferulic acid, abscisic acid, kaempferol, quercetin, naringerin, hisperidin, miricetin, quercetin, esculetin, pinocembrin Megastigmatrienone, 2-p-hydroxiphenylalcohol, β-pinene, linalool oxide	[3,24,26,34]
*Gevuina avellana* (Chilean hazelnut)	*Escherichia coli*, *P. aeruginosa*, *S. aureus*, *Streptococcus pyogenes*	Determined by TPC and FRAP assays	None reported	Acetofenone	[24]
*Escallonia pulverulenta* (corontillo)	None reported	None reported	None reported	Derivatives from catechin gallic acid, protocatehuic acid, 2,4-di-hydroxibenzoic acid, catechin, epicatechin, p-coumaric acid quercetin, kaempferol safranal, hotrienol, trans β-damascenone	[4]
*Caldcluvia paniculata* (tiaca)	*Escherichia coli*, *P. aeruginosa*, *S. aureus*, *Streptococcus pyogenes*	Determined by TPC and FRAP assays	None reported	Rutin, caffeic acid, pinocembrin, chrysin	[24]
*Trevoa trinervis* (tebo o tevo)	None reported	Determined by TPC and DPPH assays	None reported	Acetone, isoamyl alcohol, acetic acid, furfural, benzaldehyde, isophorone, furfuryl alcohol, rutin, caffeic acid, pinocembrin, chrysin	[4]
*Azara* *petiolaris/* *Azara* *integrifolia* (corcolén)	None reported	Determined by TPC and ORAC assays	None reported	None reported	[28]
(*Cryptocarya* *Alba*) Peumo	None reported	None reported	None reported	Gallic acid, benzoic acid derivatives, protocatehuic acid, 2,4-di-hydroxybenzoic acid, catechin, epicatechin, rutin, ellagic acid, miricetin, quercetin, kaempferol	[4]

## References

[1] Schencke C., Vásquez B., Sandoval C., del Sol M. (2016). El rol de la miel en los procesos morfofisiológicos de reparación de heridas. Int. J. Morphol..

[2] Mejias E., Gómez C., Garrido T., Godoy P., Gómez M., Montenegro G. (2019). Natural attributes of Chilean honeys modified by the presence of neonicotinoids residues. Agrofor. Syst..

[3] Montenegro G., Salas F., Peña R.C., Pizarro R. (2009). Antibacterial and antifungic activity of the unifloral honeys of *Quillaja saponaria*, an endemic Chilean species. Phyton.

[4] FAO (2023). Mieles Chilenas para el Mundo: Atributos, propiedades e innovación. Gloria Montenegro.

[5] Norma Chilena NCh2981 (2005). Miel de Abejas-Denominación de Origen Botánico Mediante Ensayo Melisopalinológico.

[6] Lizama A.G. (2023). Boletín Interactivo Apícola. ODEPA—Oficina de Estudios y Políticas Agrarias.

[7] Rodríguez R., Marticorena C., Alarcón D., Baeza C., Cavieres L., Finot V.L., Fuentes N., Kiessling A., Mihoc M., Pauchard A. (2018). Catálogo de las plantas vasculares de Chile. Gayana Botánica.

[8] Larsson D.J., Flach C.F. (2022). Antibiotic resistance in the environment. Nat. Rev. Microbiol..

[9] Bartlett J.G., Gilbert D.N., Spellberg B. (2013). Seven ways to preserve the miracle of antibiotics. Clin. Infect. Dis..

[10] Kalidasan G., Saranraj P., Ragul V., Sivasakthi S. (2017). Antibacterial activity of natural and commercial honey—A comparative study. Adv. Biol. Res..

[11] Nolan V.C., Harrison J., Cox J.A.G. (2019). Dissecting the antimicrobial composition of honey. Antibiotics.

[12] Furman D., Campisi J., Verdin E., Carrera-Bastos P., Targ S., Franceschi C., Ferrucci L., Gilroy D.W., Fasano A., Miller G.W. (2019). Chronic inflammation in the etiology of disease across the life span. Nat. Med..

[13] Vonkeman H.E., van de Laar M.A.F.J. (2010). Nonsteroidal anti-inflammatory drugs: Adverse effects and their prevention. Semin. Arthritis Rheum..

[14] Whitehouse M.W. (2010). Anti-inflammatory glucocorticoid drugs: Reflections after 60 years. Inflammopharmacology.

[15] Pan M.-H., Lai C.-S., Ho C.-T. (2010). Anti-inflammatory activity of natural dietary flavonoids. Food Funct..

[16] Oryan A., Alemzadeh E., Moshiri A. (2016). Biological properties and therapeutic activities of honey in wound healing: A narrative review and meta-analysis. J. Tissue Viability.

[17] Alfadda A.A., Sallam R.M. (2012). Reactive oxygen species in health and disease. J. Biomed. Biotechnol..

[18] Ciappini C., Stoppani F., Martinet R., Alvarez M. (2013). Actividad antioxidante y contenido de compuestos fenólicos y flavonoides en mieles de tréboles, eucalipto y alfalfa. Rev. Cienc. Tecnol..

[19] Zeb A. (2020). Concept, mechanism, and applications of phenolic antioxidants in foods. J. Food Biochem..

[20] Alvarez-Suarez J.M., Gasparrini M., Forbes-Hernández T.Y., Mazzoni L., Giampieri F. (2014). The composition and biological activity of honey: A focus on manuka honey. Foods.

[21] Olate-Olave V.R., Guzmán L., López-Cortés X.A., Cornejo R., Nachtigall F.M., Doorn M., Santos L.S., Bejarano A. (2021). Comparison of Chilean honeys through MALDI-TOF-MS profiling and evaluation of their antioxidant and antibacterial potential. Ann. Agric. Sci..

[22] Bridi R., Montenegro G. (2017). The value of Chilean honey: Floral origin related to their antioxidant and antibacterial activities. Honey Analysis.

[23] Velásquez P., Montenegro G., Leyton F., Ascar L., Ramirez O., Giordano A. (2019). Bioactive compounds and antibacterial properties of monofloral ulmo honey. CYTA—J. Food.

[24] Montenegro G., Santander F., Jara C., Núñez G., Fredes C. (2013). Antioxidant and antimicrobial activity of unifloral honeys of plants native to Chile. Bol. Latinoam. Caribe Plant. Med. Aromat..

[25] Sherlock O., Dolan A., Athman R., Power A., Gethin G., Cowman S., Humphreys H. (2010). Comparison of the antimicrobial activity of ulmo honey from Chile and manuka honey against methicillin-resistant *Staphylococcus aureus*, *Escherichia coli* and *Pseudo-monas aeruginosa*. BMC Complement. Altern. Med..

[26] Bridi R., Nuñez-Quijada G., Aguilar P., Martínez P., Lissi E., Giordano Villatoro A., Montenegro Rizzardini G. (2017). Differences between phenolic content and antioxidant capacity of quillay Chilean honeys and their separated phenolic extracts. Cienc. Inv. Agr..

[27] Leos-Rivas C., Rivas-Morales C., García-Hernández D.G. (2016). Actividad antioxidante y toxicidad. Investigación en Plantas de Importancia Médica.

[28] Giordano A., Retamal M., Leyton F., Martínez P., Bridi R., Velásquez P., Montenegro G. (2018). Bioactive polyphenols and antioxidant capacity of *Azara petiolaris* and *Azara integrifolia* honeys. CYTA—J. Food.

[29] Van den Berg A., Van den Worm E., Quarles van Ufford H., Halkes S., Hoekstra M., Beukelman C. (2008). An *in vitro* examination of the antioxidant and anti-inflammatory properties of buckwheat honey. J. Wound Care.

[30] Ferrero-Miliani L., Nielsen O.H., Andersen P.S., Girardin S.E. (2006). Chronic inflammation: Importance of NOD2 and NALP3 in interleukin-1β generation. Clin. Exp. Immunol..

[31] Molan P., Rhodes T. (2015). Honey: A Biologic Wound Dressing. Wounds.

[32] Yang Z., Min Z., Yu B. (2020). Reactive oxygen species and immune regulation. Int. Rev. Immunol..

[33] Schencke C., Vasconcellos A., Sandoval C., Torres P., Acevedo F., del Sol M. (2016). Morphometric evaluation of wound healing in burns treated with ulmo (*Eucryphia cordifolia*) honey alone and supplemented with ascorbic acid in guinea pig (*Cavia porcellus*). Burns Trauma.

[34] Sánchez E., Piovano M., Valdés E., Young M.E., Acevedo C.A., Osorio M. (2012). Determination of antioxidant properties of 26 Chilean honeys and a mathematical association study with their volatile profile. Nat. Prod. Commun..

[35] Acevedo F., Torres P., Oomah B.D., de Alencar S.M., Massarioli A.P., Martín-Venegas R., Albarral-Ávila V., Burgos-Díaz C., Ferrer R., Rubilar M. (2017). Volatile and non-volatile/semi-volatile compounds and *in vitro* bioactive properties of Chilean ulmo (*Eucryphia cordifolia* Cav.) honey. Food Res. Int..

[36] Khatkar A., Nanda A., Kumar P., Narasimhan B. (2017). Synthesis, antimicrobial evaluation and QSAR studies of gallic acid derivatives. Arab. J. Chem..

[37] Viteri R., Giordano A., Montenegro G., Zacconi F.C. (2022). Flavonoids and triterpenes isolated from *Eucryphia cordifolia* (*Cunoniaceae*). Biochem. Syst. Ecol..

[38] Delporte C., Rodríguez-Díaz M., Cassels B.K., Máthé Á., Bandoni A. (2021). *Quillaja saponaria* Molina. Medicinal and Aromatic Plants of South America Vol. 2. Medicinal and Aromatic Plants of the World.

[39] Montenegro G., Díaz-Forestier J., Fredes C., Rodríguez S. (2013). Phenolic profiles of nectar and honey of *Quillaja saponaria* Mol. (*Quillajaceae*) as potential chemical markers. Biol. Res..

[40] Mandal M.D., Mandal S. (2011). Honey: Its medicinal property and antibacterial activity. Asian Pac. J. Trop. Biomed..

[41] Montenegro G., Velásquez P., Viteri R., Giordano A. (2021). Changes in the antibacterial capacity of ulmo honey in relation to the contribution of *Eucryphia cordifolia* pollen. J. Food Nutr. Res..

[42] Viteri R., Zacconi F., Montenegro G., Giordano A. (2021). Bioactive compounds in *Apis mellifera* monofloral honeys. J. Food Sci..

[43] Merckoll P., Jonassen T.Ø., Vad M.E., Jeansson S.L., Melby K.K. (2009). Bacteria, biofilm and honey: A study of the effects of honey on “planktonic” and biofilm-embedded chronic wound bacteria. Scand. J. Infect. Dis..

